# Buckwheat and Rosemary as Antioxidants in Soybean Oil: Comparison With Synthetic Antioxidant

**DOI:** 10.1002/fsn3.70416

**Published:** 2025-06-10

**Authors:** Behnam Alizadeh‐Salmani, Masoumeh Arab, Jalal Sadeghizadeh‐yazdi, Mojtaba Yousefi, Sara Jambarsang

**Affiliations:** ^1^ Department of Food Sciences and Technology, School of Public Health Shahid Sadoughi University of Medical Sciences Yazd Iran; ^2^ Research Center for Food Hygiene and Safety, School of Public Health Shahid Sadoughi University of Medical Sciences Yazd Iran; ^3^ Food Safety Research Center (Salt) Semnan University of Medical Sciences Semnan Iran; ^4^ Center for Healthcare Data Modeling, Department of Biostatistics and Epidemiology, School of Public Health Shahid Sadoughi University of Medical Sciences Yazd Iran

**Keywords:** antioxidant, buckwheat, oxidation stability, rosemary, soybean oil, TBHQ

## Abstract

This study examines the antioxidant potential of buckwheat and rosemary as an alternative to synthetic antioxidants in the oil industry. The methanolic extracts were analyzed for total phenol and flavonoid content, as well as antioxidant activity using the FRAP and DPPH methods. These extracts, along with the synthetic antioxidant TBHQ, were incorporated into soybean oil at varying concentrations (0 + 800, 200 + 600, 400 + 400, 600 + 200, 800 + 0 ppm (buckwheat + rosemary) and 50 and 75 ppm TBHQ) and oxidation tests conducted during 35 days storage at 60°C. Results showed that the extract of 800 ppm rosemary had the highest levels of phenolic (82.53 ± 6.39 mg GAE/g DW) and flavonoid (37.88 ± 3.08 mg RE/g DW) compounds. Antioxidant capacity of rosemary and buckwheat (800 ppm) were not significantly different from the TBHQ. Oil treatments with 800 ppm rosemary and buckwheat were more effective in postponing oxidation than the combination treatments. Oil samples with 800 ppm natural extracts were combined with synthetic antioxidant TBHQ at concentrations of 75 and 50 ppm, and oxidation indexes were evaluated. Findings demonstrated that this combination had a synergistic effect in postponing oxidation. The most effective treatment was 800 ppm buckwheat extract combined with 75 ppm of TBHQ for delaying oxidation.

## Graphical abstract

The phenolic and flavonoid content in the methanolic extract of rosemary was higher than that of buckwheat, and the free‐radical scavenging of buckwheat and rosemary at 800 ppm was not significantly different from that of TBHQ at 75 ppm. The extracts of rosemary and buckwheat at 800 ppm showed no significant difference from TBHQ at 75 ppm in oxidation index tests. The combination of rosemary and buckwheat with TBHQ exhibited a synergistic effect.

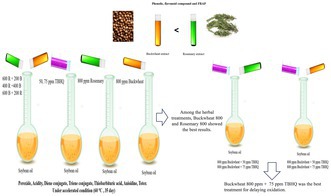

## 1 Introduction

Edible oils are considered an important category of food products. Edible oils are obtained from various plant sources such as seeds, nuts, and fruits and are widely used in cooking, food processing, and production of other consumer goods (Flores et al. 2021; Yan Zhang et al. 2023). Soybeans are the primary source of oil utilized in the food industry and account for around 60% of global oil production (Subroto et al. 2020). Soybean oil is commonly used in the food industry for various purposes, including frying, margarine manufacturing, producing trans fatty acid‐free products, and incorporating in monodiglyceride modifiers (Gerde et al. 2020). The oil content in soybeans ranges from 17% to 22% (Subroto et al. 2020), and 85% of the fatty acids in the oil are unsaturated fatty acids, among which linoleic acid has the largest share of 54.8%. Other important fatty acids in this oil include oleic acid (23.17%) and palmitic acid (11.50%; Zhao et al. 2022). Also, soybean oil contains 90%–95% of triacylglycerol compounds including phosphatides, sterols, tocopherols, and other fat‐soluble compounds. The amounts of phosphatides in this oil are about 2%, which includes lecithin and cefalin. Soybean oil includes an abundance of unsaturated molecules, including polyunsaturated fatty acids (PUFA; Subroto et al. 2020). Soybean oil exhibits a high susceptibility to oxidation as a result of its substantial content of unsaturated fatty acids.

Oxidation in food is a destructive process that occurs by forming free radicals and turning them into hydroperoxide and breaking them down into hydrocarbons such as pentane, octane, and aldehyde compounds (Martin‐Rubio et al. 2020; Wann et al. 2021). This process leads to the deterioration of nutrients and the development of undesirable odors and tastes in food products (Geng et al. 2023). The oxidative stability of oils and fats is affected by several factors such as light, metal ions, oxygen, temperature, and enzymes (Indiarto and Qonit 2020). Antioxidants are used in all kinds of oils in order to increase shelf life and maintain safety, nutritional quality, functional properties, and desirability of the products (Poljsak et al. 2021). Antioxidants are compounds that prevent the reaction of free radicals in the form of oxygen and active nitrogen with biomolecules. In addition to their roles in biological systems, in foods rich in unsaturated fats, they also prevent the reduction of nutritional qualities, undesirable tastes, and discoloration (Bensid et al. 2022; Parcheta et al. 2021). Antioxidants are divided into two categories: chemical (synthetic) and natural. Synthetic antioxidants encompass a range of compounds, namely butylated hydroxytoluene (BHT), butylated hydroxyanisole (BHA), tertiary butylhydroquinone (TBHQ), and propyl gallate (PG). Phenolic acids, flavonoids, and carotenoids are among the various types of natural antioxidants (Gulcin 2020).

Buckwheat ( *Fagopyrum esculentum* ), classified as a pseudo‐cereal, is utilized in the manufacturing of functional foods owing to its rich supplies of bioactive components (Bhinder et al. 2019). Studies show that buckwheat, compared with other grains, is rich in useful compounds, including proteins with high biological values, essential amino acids (including lysine and arginine; Zhu 2021), natural antioxidants (tocopherol and acid phenolics; Singh et al. 2024), flavonoids (rutin, hyperside, vitexin), dietary fibers, vitamins (especially B2, B1), and minerals (magnesium, lithium, iron, and potassium; Salehi et al. 2018). Buckwheat exhibits higher rutin content compared to other grains. Rutin is a flavonol compound characterized by its glycosidic structure (Kreft et al. 2022). Rutin, along with quercetin in buckwheat, has more antioxidant activities than ascorbic acid, alpha‐copherol, and beta‐carotene (Salehi et al. 2018).

Rosemary plant ( *Rosmarinus officinalis* ) belonging to the mint family *(Lumiaceae)*, is known as a valuable medicinal plant in the pharmaceutical and medical industries due to its antimicrobial and antimutagenic properties (Abada et al. 2025; Andrade et al. 2018). Rosemary also contains phenolic compounds and antioxidant activity. The most important phenolic compounds include carnosic acid, carnasol, rosmarinic acid, rosmanol, epi and isorsmanol, rosmedial and methyl carnosate (Topal and Gulcin 2022) also, compounds such as rosmarinic acid, caffeic acid, and flavonoids in rosemary, the most important of which include 4‐O‐β‐d‐glycopyranosyl acetophenone, 6‐O‐feruloyl‐glycopyranoside, lutein, and 7‐O‐β‐galactopyranoside have antioxidant properties (Micić et al. 2021).

Due to the high levels of unsaturated fatty acids in soybean oil and the toxic effects of chemical antioxidants on the body, in recent years much attention has been paid to the use of natural antioxidants. Because the fact that no research has been done on using buckwheat and rosemary as antioxidants in soybean oil, the purpose of this research was to investigate the effects of common buckwheat and rosemary extracts as natural antioxidants in reducing the oxidation process of soybean oil.

## 2 Materials and Methods

### 2.1 Materials

The buckwheat grains (*Fagopyrum escluentum*) and rosemary plant were purchased from Saghlam Company and local shops of Iran, respectively. The compounds Folin Ciocalteu reagent, sodium carbonate, gallic acid (GA), chloroform, acetic acid FeSO_4_7H_2_O, potassium iodide, thiobarbituric acid, trichloroacetic acid, phenolphthalein, isooctane, sodium hydroxide, methanol, and ethanol were purchased from Merck (Germany). P‐anisidine, diphenyl‐1‐picrylhydrazyl‐2,2 (DPPH), 2,4,6‐ tri (2‐pyridal)‐s‐triazine (TPTZ), quercetin, and rutin were obtained from Sigma‐Aldrich Co.

### 2.2 Buckwheat and Rosemary Extract Preparation

To prepare the extract, 50 g of buckwheat flour were mixed with 500 mL of 98% methanol and stirred for 24 h at a temperature of 23°C. Then it was filtered using Whatman No. 1 filter paper. The extract was concentrated by a rotary evaporator at a temperature of 38°C, and finally, the extract was dried by a vacuum dryer at a temperature of 40°C and stored in the dark at a temperature of 4°C in covered plates without air penetration (Khoshdouni 2021).

### 2.3 Characteristic of Buckwheat and Rosemary Extract

#### 2.3.1 Total Phenol Content (TPC)

Identification of phenolic compounds was done by the Folin Ciocâlteu method. After combining the extract with distilled water and 2.5 mL of Folin Ciocâlteu reagent and adding 7.5% sodium carbonate solution, the absorbance of the sample at a wavelength of 765 nm was read by a UV‐Vis spectrophotometer (hach, Germany‐ USA). Total phenolic content was determined with a calibration curve of gallic acid. Phenolic compound content was obtained as mg GAE (gallic acid equivalents)/g of DW (dry weight) (Rojas‐Ocampo et al. 2021).

#### 2.3.2 Total Flavonoid Content

Total flavonoid content was evaluated by the aluminum chloride spectrophotometric assay. Briefly, 1.5 mL of methanol, 0.1 mL of aluminum chloride (10% methanol), 0.1 mL of 1 M potassium acetate, and 2.8 mL of distilled water were added to 0.5 mL of the extract. Then, the solution was placed at room temperature for 30 min, and the absorbance of the solutions was measured at 415 nm with a spectrophotometer. Rutin was used to plot the calibration curve. The amount of flavonoid was reported based on the amount equivalent to mg of rutin equivalents (RE)/g DW (Wigati et al. 2023).

#### 2.3.3 DPPH Radical Scavenging Activity

The amount of antioxidant activity of the samples was determined based on the free‐radical scavenging activity (DPPH). 1 mL of the extract was added to 3 mL of a methanolic solution of DPPH (0.15 mm). The samples were shaken vigorously and kept for 30 min at room temperature in the dark. Then, the absorbance of the solutions was measured using a spectrophotometer at a wavelength of 517 nm, and the inhibitory activity of the extract was calculated using the following formula (Gulcin and Alwasel 2023).

$$\;\left(\%\right)\;\text{antioxidant activity}=\left(\text{A0}–A1\right)/\text{A0}\times 100$$

A0 and A1 are the absorption rate of the control sample and the irradiated sample, respectively.

#### 2.3.4 FRAP Assay

In a test tube, 1.8 mL of fresh FRAP solution was added to 200 μL of extract (FRAP solution with the addition of 25 mL of acetate buffer, 2.5 mL of TPTZ solution, and 2.5 mL of pre‐prepared mL of FeCl_3_·6 H_2_O) and was placed in the above mixture for 5 min at 37°C, and then the solution absorbance was read by a spectrophotometer at a wavelength of 595 nm. The reducing activity of samples was calculated using a standard curve in terms of mL moles of iron (II) per mg of dry weight of the sample (Sethi et al. 2020).

### 2.4 Oil Sample Preparation

In order to measure the antioxidant activity, extracts of rosemary plant and common buckwheat in different concentrations (0 + 800, 200 + 600, 400 + 400, 600 + 200, 0 + 800 mg/kg (buckwheat + rosemary)) were added to soybean oil. Also, two samples with synthetic antioxidant of TBHQ in concentrations of 50 and 75 mg/kg and one control sample without antioxidant were prepared. Then, the oil samples were kept in an oven at 60°C for 35 days, and peroxide, thiobarbituric acid, anisidine, and diene and triene conjugated tests were performed on the samples on Days 0, 7, 14, 21, 28, and 35. All the mentioned tests were done in three repetitions. After the evaluation, complex samples containing optimum concentration of natural antioxidants and TBHQ (in concentrations of 50 and 75 mg/kg) were prepared, and the oxidative stability was evaluated.

### 2.5 Oxidant Tests

#### 2.5.1 Peroxide Value

Five grams of oil was weighed and 30 mL of chloroform and acetic acid solution (3:2 ratios) and 0.5 mL of potassium iodide solution were added and placed in the dark for 1 min. Next, 30 mL of distilled water was added to the mixture with the indicator and titrated with sodium thiosulfate until a white color appeared. The peroxide number was obtained according to the following formula (Moczkowska et al. 2020):

$$\mathrm{PV}=\frac{\left(S-B\right)\times N}{G}\times 1000$$

*S*, volume of thiosulfate used in sample titration (mL); *B*, the amount of thiosulfate used in the titration of the control sample (L); *N*, normality of thiosulfate; *G*, weight of the sample (g).

#### 2.5.2 Determination of Soybean Oil Acidity

Two grams of the oil sample was weighed and 10 mL of ethanol was added to the oil sample. The amount of 0.5 mL of phenolphthalein was added and 0.1 N sodium hydroxide was titrated until a purple color was formed (Di Pietro et al. 2020). Acidity was calculated using the following formula

$$\text{Acidity}:\frac{56.1\times N\times V}{M}$$

*N*, the actual concentration of the volumetric standard of sodium or potassium hydroxide used (moles/L); *V*, volumetric standard volume of sodium or potassium hydroxide used (mL); *M*, weight of oil (g).

#### 2.5.3 2‐5‐3‐Diene and Trien Conjugate Value

To measure the conjugation of diene (DN) and trien (TN), 10 mg of oil containing the extract was mixed with 10 mL of isooctane. The absorbance was then read by a spectrophotometer at 234 and 272 nm in a quartz cell for DN and TN, respectively, against the absorbance of isooctane (as control) and calculated using the following formula (Zhang et al. 2022):

$$E=\text{A2}/\left(\mathrm{CL}\times L\right)$$

*E*, amount of micromoles of conjugated DNs or TNs per g of sample; A2, absorbance of the sample at the wavelength of 234 nm or 272; *L*, the length of the quartz cell in cm; CL, value of the sample (g).

#### 2.5.4 Determination of Thiobarbiotic Acid of Soybean Oil

One gram of oil was dissolved in 10 mL of carbon tetrachloride, and 10 mL of TBA solution (0.67% solution of thiobarbiotic acid in water mixed with the same volume of pure acetic acid) was added. Then, it was placed in a centrifuge at a speed of 1000 rpm for 5 min, its aqueous part was separated, and it was placed in a boiling water bath for 30 min. Finally, absorbance was measured at a wavelength of 532 nm. The TBA number was obtained based on the following formula (Moczkowska et al. 2020):

$${E^{18}}_{\mathrm{ICM}}=\frac{A532}{\mathrm{LM}}$$

*A*, absorbance at a wavelength of 532 nm; *L*, the length of the cuvette (cm); *M*, the weight of the sample (g).

#### 2.5.5 Anisidine Value

0.5 mL of oil sample was weighed in a 25 mL flask and made up to volume with isooctane (A). Then, 1 mL of anisidine was added to 5 mL of solution A (solution B). To prepare the standard solution, 1 mL of anisidine was added to 5 mL of isooctane. Finally, the absorption of solution A was determined at a wavelength of 350 nm by zeroing the device with isooctane as a blank, and the absorption of solution B was measured 10 min after preparation and being in the dark at the same wavelength against the standard solution as a blank, using the spectrophotometry device. The amount of anisidine was calculated according to the following formula (Moczkowska et al. 2020):

$$\mathrm{AV}=25\times \left(1.2\;\mathrm{Ab}-\mathrm{Aa}\right)/M$$

Ab, absorption of reacted solution B; Aa, absorption of unreacted solution A; *M*, weight of oil sample in g.

#### 2.5.6 Totox Number Measurement

The TOTOX number was calculated according to the following equation:

$$\text{Totox numbe}=\text{Para}-\text{anisidine number}+\left(\text{peroxide number}\times 2\right)$$

### 2.6 Statistical Analysis

In this study, the quantitative characteristics of descriptive statistics were described using the mean and standard deviation. To ensure the highest accuracy in the statistical results, the relevant tests were conducted in three repetitions. Analysis of variance (ANOVA) was used to assess the significant differences between different treatments, with a significance criterion of *p* < 0.05. Graphs were drawn using Excel and SPSS.26 software.

## 3 Result and Discussion

### 3.1 Characteristic of Buckwheat and Rosemary Extract

#### 3.1.1 Phenolic and Flavonoid Content

Phenolic and flavonoid compounds are widely recognized as potent antioxidants, significantly contributing to the antioxidant activity of various plant‐based foods and beverages. These compounds show their function by scavenging free radicals and inhibiting the conversion of hydroperoxides into free radicals (Lourenço et al. 2019; Zeb 2020). However, the antioxidant activity of phenolic and flavonoid compounds can vary considerably based on their specific chemical structures, which include factors such as the number and position of hydroxyl groups, the degree of polymerization, and other structural characteristics. Notably, not all phenolic and flavonoid compounds exhibit strong antioxidant properties; their effectiveness is contingent upon the specific compounds present and their concentrations (Hu et al. 2022). The most important phenolic compounds in rosemary include carnosic acid, carnasole, rosmarinic acid, and caffeic acid. Additionally, lutein, apigenin, and hispidolin are recognized as prominent flavonoid compounds in this herb (Mena et al. 2016). In common buckwheat, caffeic acid and vanillic acid represent important phenolic compounds, while quercetin and rutin are among the key flavonoid compounds identified (Zhong et al. 2022).

**FIGURE 1:**
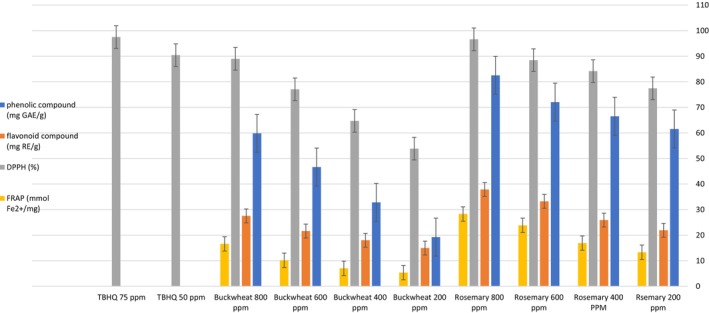
Phenolic and flavonoid concentration and antioxidant activity of rosemary and buckwheat extract and TBHQ. Different lowercase letters indicate significant differences among rosemary and buckwheat extract, and TBHQ (*p* < 0.05).

The total phenolic and flavonoid content of methanolic extracts of rosemary and buckwheat was presented in Figure 1. The findings indicated that the highest concentration of phenolic and flavonoid compounds was observed in rosemary at 800 ppm (82.53 ± 6.39 mg GAE/g DW and 37.88 ± 3.08 mg RE/gDW, respectively), while the lowest concentration was found in buckwheat at 200 ppm (19.24 ± 2.48 mg GAE/g DW and 14.96 ± 2.1 mg RE/gDW, respectively). Furthermore, it was noted that an increase in the concentration of buckwheat extract resulted in a significant elevation in the amounts of phenolic and flavonoid compounds. Various studies have reported the levels of phenolic and flavonoid compounds of buckwheat and rosemary extracts.

In one study, the methanolic extract of buckwheat contained phenolic compounds equivalent to 0.5 g of catechin equivalent to 100 g (Sun and Ho 2005). Another study reported the amounts of phenolic compounds in wheat grain, dehulled grain, and buckwheat leaves as 3303 mg/kg, 3903 mg/kg, and 39,514 mg/kg dry matter, respectively (Holasova et al. 2002). Variations in the concentrations of phenolic and flavonoid compounds in rosemary and buckwheat extracts in various studies can be ascribed to factors such as the type of raw material, the extraction methods, and the differing analytical techniques utilized for quantifying these compounds (Irakli et al. 2023; Sharma et al. 2020).

#### 3.1.2 DPPH Radical Scavenging Activity

The DPPH (2,2‐diphenyl‐1‐picrylhydrazyl) method for assessing free‐radical inhibition is widely utilized in antioxidant research, primarily due to its simplicity and high sensitivity. DPPH functions as a free radical that acts as an electron or hydrogen radical acceptor (Yasin et al. 2022). The antioxidant content of methanolic extracts of rosemary and buckwheat was presented in Figure 1. The results indicated that increasing the extract concentration caused enhanced inhibitory activity. The highest and lowest inhibitory activities were observed in rosemary at 800 ppm (%96.63 ± 2.53) and buckwheat at 200 ppm (%53.85 ± 4.20), respectively. Furthermore, the findings revealed that the inhibitory effects of 800 ppm rosemary and 800 ppm buckwheat extract did not significantly differ from those of the 50 and 75 ppm TBHQ treatments.

The DPPH free‐radical inhibitory activities of extracts can be attributed to their phenolic and flavonoid content (Amiri et al. 2023). Generally, increasing the concentration of phenolic compounds directly enhances the inhibitory power against free radicals. This enhancement occurs because a higher concentration of phenolic compounds leads to an increased number of hydroxyl groups in the reaction medium, thereby elevating the likelihood of hydrogen donation to free radicals, which subsequently increases the extract's inhibitory capacity (Becerril‐Sánchez et al. 2021; Shi et al. 2022). The free‐radical inhibitory activity of flavonoids was similarly contingent upon the presence of hydroxyl groups (Fu et al. 2021; Shen et al. 2022) Numerous studies have demonstrated that the DPPH free‐radical inhibitory activity of plant extracts is concentration‐dependent; it was found that the free‐radical inhibition effect of TBHQ exceeded that of rosemary and buckwheat extract (Sun and Ho 2005).

#### 3.1.3 FRAP Assay

The iron reduction method is a quick and suitable assay for measuring the reducing power and can be used as an indicator of the antioxidant properties of chemical compounds. When a Fe^3+^‐TPTZ complex is reduced to the Fe^2+^ form by an antioxidant under acidic conditions, an intense blue color with an absorption maximum at 593 nm is formed. Therefore, the antioxidant effect can be evaluated by monitoring the formation of the Fe^3+^‐TPTZ complex using a spectrophotometer (Sadowska‐Bartosz and Bartosz 2022).

The antioxidant values of rosemary and buckwheat extracts were shown in Figure 1. The results indicated that 200 ppm buckwheat treatment had the lowest (5.36 ± 2.03 mmol Fe^2+^/mg DW) and the 800 ppm rosemary treatment had the highest (28.28 ± 4.42 mmol Fe^2+^/mg DW) amount of FRAP. The value increased with rising extract concentration. As previously mentioned, phenolic and flavonoid compounds possess antioxidant activities and play a crucial role in mitigating oxidative stress caused by the reactivity of free radicals. The results of the current study indicated that an increase in the concentrations of buckwheat and rosemary extracts correlated with enhancing iron reduction power. This enhancement was attributed to the concomitant increase in the levels of phenolic and flavonoid compounds as the concentration of the extracts increased (Huyut et al. 2017).

In a study, the amount of FRAP in methanolic extract of buckwheat flour and grain was reported as 13 mmol Fe^2+^/mg DW (42), while this amount in ethanolic and methanolic extract of rosemary was 45.11 ± 1.129 and 114.3 ± 12.36 mmol Fe^2+^/mg DW, respectively (Al‐jaafreh 2024). In another study, this amount was reported as 54.1186 (bmM/g) for rosemary methanolic extract (Rocío Teruel et al. 2015). This variation can be justified by the differences in the type of rosemary plant, the extraction method, and the amount of phenolic and flavonoid compounds, which finally lead to different antioxidant activities (Nieto et al. 2018; Wojeicchowski et al. 2020).

### 3.2 Peroxide Value

Hydroperoxides are the primary products formed during the oxidation of fats and oils. Generally, a high degree of unsaturation in oils increases their susceptibility to oxidation. When the concentration of peroxides reaches a certain threshold, various chemical changes occur, leading to the formation of volatile aldehydes and ketones, which are secondary oxidation products responsible for the unpleasant odors and flavors associated with the deterioration of fatty substances. In the initial stages of the oxidation process, the levels of these compounds remain low; however, during the releasing phase, the concentration of hydroperoxides increases rapidly (Geng et al. 2023; Shahidi and Hossain 2022).

The results of the peroxide index are shown in Table 1. The results indicated that the peroxide index of all treatments increased significantly at the end of the storage period, with the highest and lowest peroxide index levels observed in Treatment 1 (control) and Treatment 3 (75 ppm TBHQ), respectively. However, no significant difference was observed between Treatment 3 and Treatment 8 (rosemary 800 ppm). Among the natural antioxidants, Treatments 7 (800 ppm rosemary) and 8 exhibited lower peroxide index values. The peroxide index of Treatment 7 did not differ significantly from that of Treatment 2 (50 ppm TBHQ). Additionally, it was observed that the peroxide index of Treatment 8 was significantly lower than Treatment 2, while it was significantly higher than Treatment 3. The peroxide index of the combined treatments of buckwheat and rosemary (Treatments 4, 5, and 6) was significantly higher compared to others. Among the combined treatments of natural antioxidants, Treatment 4 (400 ppm rosemary + 400 ppm buckwheat) exhibited the highest peroxide index, while Treatment 6 displayed the lowest.

**TABLE 1:** Primary oxidation of methanol extract of rosemary, buckwheat, and synthetic antioxidant TBHQ.

Parameters	Days	Treatments							
		Control	TBHQ50 ppm	TBHQ 75 ppm	Ros400 + Buck400	Ros 200 + Buck600	Ros600 + Buck200	Ros800	Buck800
Proxide (meq/kg)	0	1.48 ± 0.0^F^	1.48 ± 0.0^F^	1.48 ± 0.0^F^	1.48 ± 0.0^E^	1.48 ± 0.0^F^	1.48 ± 0.0^F^	1.48 ± 0.0^F^	1.48 ± 0.0^F^
	7	8 ± 0.08^aE^	3.2 ± 0.08^dE^	2.94 ± 0.03^eE^	6 ± 0.01^bD^	4.94 ± 0.04^cE^	5.95 ± 0.03^aE^	1.96 ± 0.06^fE^	2 ± 0.04^fE^
	14	14.81 ± 0.032^aD^	7 ± 0.09^dD^	5.94 ± 0.02^efD^	9.73 ± 0.56^bC^	8.83 ± 0.032^cD^	8.93 ± 0.04^cD^	6.92 ± 0.043^dD^	7 ± 0.19^dD^
	21	18.81 ± 0.05^aC^	9.85 ± 0.03^eC^	7.97 ± 0.03^hC^	12.21 ± 0.04^bC^	11.37 ± 0.05^cC^	10.86 ± 0.041^dC^	8.01 ± 0.051^gC^	8.88 ± 0.061^fC^
	28	26.67 ± 0.064^aB^	12.2 ± 0.03^eB^	10.21 ± 0.05^gB^	16.76 ± 0.061^bB^	14.46 ± 0.07^cB^	13.88 ± 0.06^dB^	10.49 ± 0.08 ^gB^	10.94 ± 0.14^fB^
	35	34.5 ± 0.1^aA^	17.9 ± 0.07^eA^	14.24 ± 0.08^gA^	21.36 ± 0.052^bA^	19.75 ± 0.04^cA^	18.88 ± 0.02^dA^	14.81 ± 0.09^gA^	15 ± 0.07^fA^
Acidity (mgKOH/g)	0	0.1 ± 0^E^	0.1 ± 0^E^	0.1 ± 0^E^	0.1 ± 0^E^	0.1 ± 0^E^	0.1 ± 0^E^	0.1 ± 0^E^	0.1 ± 0^E^
	7	0.29 ± 0.026^aE^	0.15 ± 0.015^dcE^	0.14 ± 0.01^dcE^	0.19 ± 0.002^dcE^	0.24 ± 0.02^bE^	0.24 ± 0.015^bE^	0.15 ± 0.02^dcE^	0.15 ± 0.015^dcE^
	14	1.72 ± 0.15^aD^	0.85 ± 0.05^eD^	0.64 ± 0.04^gfD^	1.51 ± 0.056^bD^	1.21 ± 0.036^cD^	1.08 ± 0.041^dD^	0.74 ± 0.04^feD^	0.84 ± 0.072^eD^
	21	3.04 ± 0.05^aC^	1.49 ± 0.055^dC^	1.35 ± 0.045^eC^	2.21 ± 0.03^bC^	1.95 ± 0.045^cC^	1.98 ± 0.041^cC^	1.4 ± 0.045^edC^	1.44 ± 0.05^edC^
	28	4.99 ± 0.1^aB^	3.29 ± 0.04^bcdB^	2.61 ± 0.036^fB^	3.56 ± 0.21^bB^	3.65 ± 0.05^bcB^	3.53 ± 0.081^bB^	3.88 ± 1.7^cdeB^	3.09 ± 0.065^deB^
	35	7.08 ± 0.026^aA^	5.6 ± 0.04^dA^	4.94 ± 0.04^gA^	6.08 ± 0.41^bA^	5.87 ± 0.092^cA^	5.75 ± 0.04^cA^	5.3 ± 0.06^fA^	5.09 ± 0.03^eA^

*Note:* Different lowercase letters indicate a significant difference between rosemary and buckwheat extracts (*p* < 0.05). Different capital letters indicate significant differences between different days (*p* < 0.05).

Various studies have demonstrated that the use of natural antioxidants in combination may result in antagonistic effects (Chen et al. 2022). These effects can be attributed to the factors such as mutual interactions, differences in chemical structure and activity, and concentration dependence (Olszowy‐Tomczyk 2020; Olszowy et al. 2019; Skroza et al. 2022). Antioxidants may interact in ways that diminish their overall effectiveness, as they can compete for similar reactive species (free radicals). This interaction can produce an antagonistic effect, whereby the combined efficacy is less than the sum of their individual effects (Olszowy et al. 2019). Additionally, the type of complex formed between two antioxidants can further diminish the antioxidant capacity of each other, contributing to the antagonistic outcome (Neunert et al. 2015; Tavadyan and Minasyan 2019). The concentration of antioxidants is a crucial factor in determining their effectiveness. Several studies have demonstrated that the relationship between concentration and antioxidant activity is not always linear (Fernandes et al. 2016). An increase in the concentration of one antioxidant may influence the antioxidant capacity of another compound, as the competition to inhibit free radicals intensifies (Naksuriya and Okonogi 2015; Palma et al. 2017; Vicol et al. 2024). Higher peroxide index of combined treatments of rosemary and buckwheat suggesting an antagonistic effect between these antioxidants. Previous studies have also demonstrated antagonistic effects of rutin and rosmarinic acid (Hajimehdipoor et al. 2014), as well as caffeic acid and quercetin (Abou Samra et al. 2011; Peyrat‐Maillard et al. 2003). Additionally, storage time significantly affects the antioxidant capacity of compounds, which various studies showed a decrease in antioxidant activity over time (Sik et al. 2021; Wang et al. 2016). It was mentioned that reduction of antioxidant capacity of rosmarinic acid over time was more pronounced than quercetin (Choulitoudi et al. 2021). Temperature is another critical factor influencing antioxidant capacity. Researches have shown that quercetin and rutin of common buckwheat exhibit greater thermal resistance compared to rosmarinic acid, carnasol, and rosmanol present in rosemary (Bhatia et al. 2022; Zhang et al. 2012).

### 3.3 Acidity Values

Acidity, as measured by free fatty acid content, increases during the oxidation of fats and oils due to the hydrolysis of triglycerides and the subsequent release of free fatty acids (Chammem et al. 2015). This process leads to a reduction in both the unsaturation and chain length of fatty acids, thereby altering their functional properties (Nitbani et al. 2020; Tan et al. 2021) Antioxidants such as rosemary and buckwheat can mitigate these oxidative changes by neutralizing reactive oxygen species through hydrogen donation to free radicals, thus preserving the integrity of fatty acids.

The results of the acidity index are shown in Table 1. The results indicated that the acidity index significantly increased after 7 days. During the 35 days, the control sample exhibited the highest (7.08 ± 0.026 mgKOH/g) and Treatment 7 demonstrated the lowest (5.3 ± 0.06 mgKOH/g) acidity. The acidity indices of Treatments 7 and 8 were significantly lower than that of Treatment 2, yet significantly higher than that of Treatment 3. Among the combined treatments of rosemary and buckwheat, Treatment 6 (600 ppm rosemary + 200 ppm buckwheat) exhibited the lowest acidity level, although it remained higher than those of the treatments containing synthetic antioxidants. Samples containing buckwheat and rosemary at a concentration of 800 ppm were found to be more effective in preventing the formation of free fatty acids.

At high temperatures, soybean oil undergoes oxidation. Oxidation breaks down triglycerides into various compounds, including free fatty acids. Free radicals produced by oxidation can initiate chain reactions, further degrading the oil and increasing acidity. Over time and at high temperatures, antioxidants are degraded and lose their properties, thus increasing the rate of oil oxidation. It was reported that the peroxide values in soybean oil increase from 0.53 mEq.O2/kg to 94.42 mEq.O2/kg after 21 days at 60°C, even in the presence of antioxidants such as olive leaf extract (Zahran and Najafi 2020). Previous research demonstrated that rosemary extract effectively inhibited the formation of free fatty acids in sunflower oil at 60°C over a period of 21 days (Chen et al. 2014) and palm oil at a temperature of 65°C (Guo et al. 2016) which aligns with the findings of the present study. Additionally, another study found that the rosemary extract successfully reduced the acidity of hazelnut oil (Tohma and Turan 2015) and hemp seed oil (Moczkowska et al. 2020).

### 3.4 Diens and Triens Conjugated

During the oxidation process, the positions of double and triple bonds shift, resulting in increasing the concentrations of DN and TN that are important compounds for investigating the initial stages of oxidation (Zubairee et al. 2025). By giving hydrogen to free radicals and preventing the formation of hydroperoxides, antioxidants prevent the transfer of double and triple bonds and in this way reduce the conjugated DN and TN index. Various research has shown that antioxidants can react with electrophilic centers in oxidized fatty acids and lead to a decrease in their conjugation level (Bonner and Arbiser 2014).

The results of conjugated DN and TN index are shown in Table 2. The results indicated that the levels increased significantly during the storage period, with a marked rise observed on Day 14. At the end of the storage, the levels in the control sample were significantly higher than those in other treatments. Among the treatments with natural antioxidants, Treatments 7 and 8 showed the lowest levels of conjugated DN and TN. Both were significantly lower than Treatment 2 while being significantly higher than Treatment 3. Among the combined treatments of buckwheat and rosemary, Treatments 5 and 6 did not show significant differences compared to Treatment 2 but exhibited higher DN and TN levels than Treatment 3. The concentration of DN and TN is directly correlated with the extent of oxidation. Conversely, the generation of free radicals and free fatty acids facilitated the migration of double bonds. The findings indicated that over time (between Days 28 and 35), the degradation of antioxidants and their diminishing efficacy led to a significant increase in the levels of DN and TN. Notably, samples supplemented with antioxidants exhibited lower indices of DN and TN compared to those without antioxidants.

**TABLE 2:** Dien conjugate value of methanol extract of rosemary, buckwheat, and synthetic antioxidant TBHQ.

Parameters	Days	Treatments							
		Control	TBHQ50 ppm	TBHQ 75 ppm	Ros400 + Buck400	Ros 200 + Buck600	Ros600 + Buck200	Ros800	Buck800
Dien conjugate (μmol/g)	0	0.1 ± 0^F^	0.1 ± 0^E^	0.1 ± 0^E^	0.1 ± 0^F^	0.1 ± 0^F^	0.1 ± 0^E^	0.1 ± 0^E^	0.1 ± 0^E^
	7	1.26 ± 0.07^aE^	0.5 ± 0.07^cD^	0.37 ± 0.04^cdE^	0.84 ± 0.06^bE^	0.72 ± 0.02^bE^	0.79 ± 0.1^bE^	0.28 ± 0 0.11^cdE^	0.33 ± 0.12^cdE^
	14	2.13 ± 0.07^aD^	1.2 ± 0.06^dD^	0.82 ± 0.05^efD^	1.76 ± 0.06^bD^	1.54 ± 0.04^bcD^	1.6 ± 0.07^cD^	1.23 ± 0.04^dD^	1.3 ± 0.035^dD^
	21	4.08 ± 0.13^aC^	2.66 ± 0.061 ^d C^	1.57 ± 0.08^hiC^	3.2 ± 0.02^bC^	3 ± 0.08^bcC^	2.84 ± 0.07^cdC^	2.17 ± 0.11^eC^	2.19 ± 0.03^eC^
	28	5.88 ± 0.092^aB^	4.15 ± 0.07^eB^	2.44 ± 0.1^ijB^	4.99 ± 0.07^bB^	4.73 ± 0.041^cB^	4.44 ± 0.11^dB^	3.16 ± 0.06^fB^	3.2 ± 0.06^fB^
	35	8.2 ± 0.03^aA^	7.3 ± 0.07^cA^	6.78 ± 0.051^fgA^	7.67 ± 0.22^bA^	7.63 ± 0.05^bcA^	7.51 ± 0.09^bcA^	6.83 ± 0.05^dA^	7.05 ± 0.05^deA^
Trien conjugate (μmol/g)	0	0.01 ± 0^F^	0.01 ± 0^F^	0.01 ± 0^F^	0.01 ± 0^E^	0.01 ± 0^F^	0.01 ± 0^E^	0.01 ± 0^E^	0.01 ± 0^D^
	7	0.35 ± 0.11^aE^	0.093 ± 0.008^cdE^	0.057 ± 0.003^efE^	0.13 ± 0.008^bE^	0.094 ± 0.002^cE^	0.13 ± 0.011^bD^	0.026 ± 0.008^gD^	0.034 ± 0.001^fgD^
	14	1.07 ± 0.015^aD^	0.45 ± 0.018^dD^	0.028 ± 0.011^efD^	0.7 ± 0.011^bD^	0.5 ± 0.014^cdD^	0.55 ± 0.023^cD^	0.43 ± 0.023^dD^	0.45 ± 0.017^dC^
	21	2.5 ± 0.057^aC^	1.74 ± 0.017^eC^	0.074 ± 0.008^hC^	2.1 ± 0.034^bC^	1.95 ± 0.017^bcC^	1.85 ± 0.043^dC^	1.39 ± 0.037^fC^	1.56 ± 0.033^eB^
	28	3.98 ± 0.039^aB^	2.96 ± 0.066^bcD^	1.88 ± 0.034^deB^	3.21 ± 0.059^bB^	3.09 ± 0.051^bcC^	3.04 ± 0.24^bcB^	2.29 ± 0.046^dB^	2.5 ± 0.023^cdA^
	35	5.58 ± 0.71^aA^	4.6 ± 0.046^cA^	4.09 ± 0.052^dA^	4.96 ± 0.058^bA^	4.8 ± 0.029^bcA^	4.72 ± 0.069^bcA^	4.14 ± 0.075^deA^	4.26 ± 0.13^dA^

*Note:* Different lowercase letters indicate a significant difference between rosemary and buckwheat extracts (*p* < 0.05). Different capital letters indicate significant differences between different days (*p* < 0.05).

Previous investigation found that ethanolic extract of rosemary at a concentration of 1000 mg exhibited a lower efficacy in reducing the formation of conjugated DN compared to TBHQ at a concentration of 100 mg in soybean oil at 63°C (Cordeiro et al. 2013). While other studies indicated that the rosemary extract effectively reduced the formation of conjugated DN in soybean oil (Samotyja and Małecka 2010) and sunflower and soybean oils (Chammem et al. 2015), this is consistent with the findings of the current study.

### 3.5 Thiobarbituric Acid Value

The thiobarbituric acid (TBA) test, which quantifies the amount of malondialdehyde (MDA) in oil, serves as a key indicator for measuring secondary oxidation products. Typically, the concentration of TBA is low in the initial stages of storage; as oxidation advances and peroxide levels increase, hydroperoxides decompose into smaller compounds such as alcohols, aldehydes, free fatty acids, and ketones. This decomposition results in elevated TBA levels. Antioxidants notably extend the induction period before lipid oxidation, thereby lowering the TBA index during storage. Moreover, research has shown that certain antioxidants may interact with secondary oxidation products like MDA, thus limiting their reactivity with TBA. This interaction highlights the necessity of selecting suitable antioxidants to improve the oxidative stability of lipid‐containing products (Miguel 2010; Mollica et al. 2020). The results of the TBA index are shown in Table 3. The results indicated that the TBA index increased significantly over time. The control sample exhibited the highest TBA level (8.99 ± 0.07 mg MLD/g), while Treatment 3 showed the lowest level (5.11 ± 0.12 mg MLD/g). In contrast, among the treatments with natural antioxidants, Treatments 7 and 8 exhibited the lowest levels of TBA. Notably, Treatments 7 and 8 did not show a significant difference compared to Treatment 3. Temperature accelerates the oxidation of polyunsaturated fatty acids (PUFAs) such as linoleic (18:2) and linolenic (18:3) acids (Benbouriche et al. 2022). Temperature increases radical chain reactions that lead to the formation of hydroperoxides and subsequent degradation to MDA, thereby increasing the TBA index. On the other hand, various studies have shown a direct relationship between the TBA index and the indices of conjugated DN and TN, and the increase of each affects the other (Padehban et al. 2018; Srivastava and Semwal 2015). The high PUFA content of soybean oil makes it particularly vulnerable to thermal oxidation. Gas chromatography (GC‐FID) analysis shows that linoleic and linolenic acids decompose rapidly under heat, directly increasing the level of MDA and, consequently, the TBA index. The present study showed that over time, the amount of MDA was formed at a higher rate (Days 28 and 35) (Benbouriche et al. 2022; Ravi Kiran et al. 2015). It was found that the methanolic extract of rosemary exhibited a lower TBA index compared with the synthetic antioxidant butylated hydroxytoluene (BHT) after 14 days of storage at 60°C in hemp seed oil (Moczkowska et al. 2020); however, in another study, the methanolic extract of rosemary showed higher levels of thiobarbituric acid than TBHQ in sunflower oil (Chen et al. 2014). These findings are consistent with the results of the current study.

**TABLE 3:** Tiobabitoric acid value of methanol extract of rosemary, buckwheat, and synthetic antioxidant TBHQ.

Parameters	Days	Treatments							
		Control	TBHQ50 ppm	TBHQ 75 ppm	Ros400 + Buck400	Ros 200 + Buck600	Ros600 + Buck200	Ros800	Buck800
Tiobarbitoric acid (mg MLD/g)	0	0.07 ± 0^E^	0.07 ± 0^E^	0.07 ± 0^D^	0.07 ± 0^D^	0.07 ± 0^F^	0.07 ± 0^E^	0.07 ± 0^E^	0.07 ± 0^E^
	7	0.87 ± 0.081^Ae^	0.55 ± 0.041^bcdD^	0.47 ± 0.026^fedcD^	0.47 ± 0.026^dA^	0.6 ± 0.02^bcE^	0.71 ± 0.02^abE^	0.27 ± 0.092^fghE^	0.35 ± 0.05^ghE^
	14	1.92 ± 0.1^ad^	1.9 ± 0.17^dD^	0.9 ± 0.1^efD^	0.9 ± 0.1^cC^	1.48 ± 0.09^cD^	1.56 ± 0.09^cD^	1.16 ± 0.091^deD^	1.2 ± 0.02^dD^
	21	3.7 ± 0.07^aC^	2.48 ± 0.172^cC^	1.41 ± 0.083^hiC^	1.41 ± 0.083^bcC^	2.81 ± 0.1^aC^	2.7 ± 0.081_bcC_	1.88 ± 0.072^efC^	2.2 ± 0.1^Dc^
	28	6.4 ± 0.1^aB^	3.4 ± 0.09^cdB^	2.76 ± 0.0^fgB^	2.76 ± 0.07^bB^	3.81 ± 0.02^bcB^	3.67 ± 0.07^bcB^	2.93 ± 0.35^efgB^	3.23 ± 0.041^edB^
	35	8.99 ± 0.07^aA^	5.3 ± 0.12^dA^	5.11 ± 0.12^cdeA^	6.1 ± 0.1^aA^	5.9 ± 0.051^bA^	5.67 ± 0.064^cA^	5.14 ± 0.14^defA^	5.27 ± 0.08^deA^

*Note:* Different lowercase letters indicate a significant difference between rosemary and buckwheat extracts (*p* < 0.05). Different capital letters indicate significant differences between different days (*p* < 0.05).

### 3.6 Anisidine Value

P‐anisidine (AnV) plays an important role in the oxidation process of edible oils. Calculation of AnV is one of the oldest methods to evaluate the secondary oxidation of oil. The results of the anisidine index are shown in Table 4. The results indicated that the anisidine index of all treatments increased significantly after 14 days. The control sample exhibited the highest, and Treatment 3 had the lowest anisidine levels. Treatments 7 and 8 were not significantly different from Treatment 3. Among the combined treatments, Treatment 4 recorded the highest anisidine index. Additionally, Treatment 6 did not differ significantly from Treatment 2.

**TABLE 4:** Anisidine value of methanol extract of rosemary, buckwheat, and synthetic antioxidant TBH.

Parameters	Days	Treatments							
		Control	TBHQ50 ppm	TBHQ 75 ppm	Ros400 + Buck400	Ros 200 + Buck600	Ros600 + Buck200	Ros800	Buck800
Anisidine (Mg/g)	0	0.54 ± 0^E^	0.54 ± 0^E^	0.54 ± 0^E^	0.54 ± 0^F^	0.54 ± 0^F^	0.54 ± 0^E^	0.54 ± 0^E^	0.54 ± 0^E^
	7	1.79 ± 0.2^aE^	1.09 ± 0.11^cdE^	0.84 ± 0.11^deE^	1.64 ± 0.11^aE^	1.24 ± 0.07bcE	1.54 ± 0.16^abE^	0.64 ± 001^eE^	0.77 ± 0.07^edE^
	14	3.96 ± 0.15^aD^	2.7 ± 0.07^eD^	1.88 ± 0.1^gD^	3.61 ± 0.032^bD^	3.2 ± 0.19^cdD^	3.28 ± 0.14^bcB^	2.7 ± 0.08^efD^	2.46 ± 0.17^eD^
	21	5.96 ± 0.087^aC^	4.28 ± 0.11^cdC^	3.16 ± 0.081^gC^	5.08 ± 0.07^bDC^	4.85 ± 0.141^bC^	4.6 ± 0.19^bcC^	3.81 ± 0.01^defgC^	3.67 ± 0.16^edC^
	28	8.99 ± 0.07^aB^	6.44 ± 0.1d^eB^	6.24 ± 0.06^efB^	7.4 ± 0.08^bB^	7.47 ± 0.16^bB^	6.75 ± 0.061^dcB^	6.73 ± 0.1^dB^	6.5 ± 0.05^deB^
	35	15.31 ± 0.1^aA^	11.23 ± 0.05^edA^	10.25 ± 0.11^gA^	12.1 ± 0.1^bA^	11.86 ± 0.06^bcA^	11.58 ± 0.091^dcA^	10.49 ± 0.47^gA^	10.33 ± 0.043^gA^

*Note:* Different lowercase letters indicate a significant difference between rosemary and buckwheat extracts (*p* < 0.05). Different capital letters indicate significant differences between different days (*p* < 0.05).

As storage duration and temperature increase, particularly under conditions such as 60°C, the decomposition of the abundant unsaturated fatty acids in soybean oil accelerates. Consequently, the rate of hydroperoxide formation and their subsequent conversion into secondary compounds, including aldehydes, increase, leading to an elevated anisidine index (Lee et al. 2021). Furthermore, over an extended storage period of 28–35 days, the antioxidant capacity of the extracts, as well as that of the synthetic antioxidant TBHQ, diminishes, resulting in a more rapid formation of aldehydes (Mansour et al. 2022). Various studies have shown that rosemary had a strong ability in preventing the formation of secondary oxidation products such as anisidine in sunflower and soybean oil (Chen et al. 2014; Dias et al. 2015; Samotyja and Małecka 2010).

### 3.7 Totox Value

The Totox index is a valuable oxidation indicator because it provides a complete view of the entire oil oxidation reaction, including the effects of early (peroxide) and late (anisidine) oxidation steps. The results of the Totox index are shown in Table 5. The Totox index provides a more comprehensive view of oil quality than peroxide or anisidine alone because a higher Totox value indicates that the oil has undergone significant oxidative changes, which makes such oil unsuitable for consumption. In various analyses, oils with low primary peroxides and anisidines had low Totox values, indicating high quality. Conversely, oils stored improperly or for long periods showed much higher Totox values due to high oxidation (Memon et al. 2022; Roshni 2019).

**TABLE 5:** Totox value of methanol extract of rosemary, buckwheat, and synthetic antioxidant TBHQ.

Parameters	Days	Treatments							
		Control	TBHQ50 ppm	TBHQ 75 ppm	Ros400 + Buck400	Ros 200 + Buck600	Ros600 + Buck200	Ros800	Buck800
TOTOX	0	3.5 ± 0^F^	3.5 ± 0^F^	3.5 ± 0^F^	3.5 ± 0^E^	3.5 ± 0^F^	3.5 ± 0^F^	3.5 ± 0^E^	3.5 ± 0^E^
	7	17.79 ± 0.36^aE^	7.49 ± 0.27^dE^	6.72 ± 0.17^eE^	13.65 ± 0.13^bD^	11.14 ± 0.15^cE^	13.45 ± 0.22^bE^	4.56 ± 0.22^gE^	4.77 ± 0.15^gE^
	14	33.59 ± 0.18^aD^	16.7 ± 0.25^dD^	13.08 ± 0.14^gD^	23.08 ± 1.16^bfC^	20.87 ± 0.25^cD^	21.14 ± 0.21^cD^	16.54 ± 0.16^edD^	16.46 ± 0.55^dD^
	21	42.32 ± 0.18^aC^	23.98 ± 0.17^eC^	17.21 ± 0.14^iC^	31.51 ± 0.15^bC^	27.59 ± 0.24^cC^	26.33 ± 0.27^dC^	19.84 ± 0.11^gC^	21.45 ± 0.28^fC^
	28	62.34 ± 0.19^aB^	30.84 ± 0.16^eB^	25.74 ± 0.16^hB^	40.9 ± 0.2^bB^	36.4 ± 0.3^cB^	34.52 ± 0.18^dB^	27.71 ± 0.26^fB^	28.42 ± 0.33^fB^
	35	84.3 ± 0.3^aA^	47.04 ± 0.19^eA^	38.73 ± 0.28^fA^	54.82 ± 0.19^bA^	51.36 ± 0.14^cA^	49.34 ± 0.11^dA^	39.71 ± 0.65^fA^	40.3 ± 0.18^fA^

*Note:* Different lowercase letters indicate a significant difference between rosemary and buckwheat extracts (*p* < 0.05). Different capital letters indicate significant differences between different days (*p* < 0.05).

The results indicated that the Totox index increased significantly after 7 days in all treatments. The control treatment exhibited the highest, and Treatment 3 demonstrated the lowest. The Totox index of Treatments 7 and 8 was not significantly different from Treatment 3. All combined treatments had a higher Totox index compared with those containing synthetic antioxidants. Previous studies investigated the antioxidant effect of rosemary extract in soybean oil during 20 days of storage at 60°C (Dias et al. 2015), sunflower oil at 100°C for 30 days (Sahunie 2024) and combined oil of perilla seed oil and palmolein at 60°C for 30 days (Kumari Singh et al. 2024) and found that the Totox index increased significantly over time, which was similar to the results of the present study.

### 3.8 Evaluation of Optimum Treatment in Combination With TBHQ

As cleared in previous sections, Treatments 7 and 8 were the more sufficient treatments in preventing oxidation. These treatments were combined with TBHQ at concentrations of 50 and 75 ppm (Treatments 9–12), and oxidation tests were measured under accelerated conditions (60°C and 35 days; Table 6). Results cleared that for all indices, Treatment 12 (800 ppm rosemary + 75 ppm TBHQ) showed the lowest, and Treatment 9 (800 ppm rosemary + 50 ppm TBHQ) presented highest levels. The findings suggested a synergistic effect between common buckwheat, rosemary, and TBHQ. The synergistic interactions among antioxidants can occur through three mechanisms: hydrogen atom transfer, simultaneous electron transfer, and complexation (Olszowy‐Tomczyk 2020). In the complexation mechanism, rosemary and buckwheat, in conjunction with TBHQ, have the capacity to chelate metal ions. In terms of the hydrogen atom transfer mechanism, rosemary and buckwheat combined with TBHQ can release hydrogen ions and inhibit free radicals produced in the chain reactions of oxidation of fats and oils. Additionally, phenolic compounds have the ability to restore the antioxidant capacity of TBHQ. Both rosemary and buckwheat extracts are rich in phenolic antioxidants and can replenish TBHQ by transferring protons. Gongling et al. (2016) demonstrated that the addition of rosemary extract and TBHQ to radish seed oil resulted in significant improvements in antioxidant capacity at 60°C. The findings indicated a synergistic effect between rosemary and TBHQ (Gongling et al. 2016). Similarly, Martinez at al (2013) reported analogous results in walnut samples (Martínez et al. 2013). Among the combined treatments of rosemary and buckwheat with TBHQ, buckwheat exhibited a stronger synergistic effect than rosemary when paired with TBHQ. As mentioned in the peroxide section, various factors, including temperature, concentration, time duration, and complex formation, contributed to a more pronounced synergistic effect of buckwheat with TBHQ compared to rosemary.

**TABLE 6:** Combined treatments of rosemary and buckwheat with TBHQ.

Parameters	Days	Treatments			
		Ros 800 ppm + TBHQ 50 ppm	Rose 800 + TBHQ 75 ppm	Buck 800 + TBHQ 50 ppm	BUCK 800 ppm + TBHQ 75 ppm
Proxide (meq/kg)	0	1.48 ± 0.0^F^	1.48 ± 0.0^E^	1.48 ± 0.0^F^	1.48 ± 0.0^F^
	7	2.57 ± 0.03^eE^	1.96 ± 0.081^fE^	2.96 ± 0.041^eE^	1.98 ± 0.015^fE^
	14	6 ± 0.08^efD^	5.53 ± 0.11^fD^	5.93 ± 0.1^efD^	5.93 ± 0.09^efD^
	21	7.5 ± 0.14^gC^	6.57 ± 0.04^jC^	7.34 ± 0.02^hC^	7.02 ± 0.11^iC^
	28	10.1 ± 0.03^gB^	9.2 ± 0.04^jB^	9.94 ± 0.07^hiB^	9.72 ± 0.07^hiB^
	35	14.05 ± 0.11^hA^	13.33 ± 0.13^Ia^	13.94 ± 0.05^iA^	13.98 ± 0.11^hA^
Acidity (mgKOH/g)	0	0.1 ± 0^E^	0.1 ± 0^E^	0.1 ± 0^F^	0.1 ± 0^E^
	7	0.12 ± 0.004^dE^	0.156 ± 0.01^dcE^	0.167 ± 0.004^dcE^	0.15 ± 0.005^dcE^
	14	0.57 ± 0.03^gD^	0.63 ± 0.005^fgD^	0.6767 ± 0.032^gD^	0.71 ± 0.036^fD^
	21	1.21 ± 0.036^fC^	1.31 ± 0.035^feC^	1.35 ± 0.05^cC^	1.39 ± 0.036^edC^
	28	2.46 ± 0.06^fB^	2.73 ± 0.09^efB^	2.76 ± 0.036^efB^	2.85 ± 0.045^efB^
	35	4.48 ± 0.0527 ^hA^	4.61 ± 0.035^hA^	4.67 ± 0.036^hA^	4.85 ± 0.045^gA^
Diene conjugates (μmol/g)	0	0.1 ± 0^D^	0.1 ± 0^D^	0.1 ± 0^E^	0.1 ± 0^E^
	7	0.18 ± 0.01^dD^	0.3 ± 0.04^dcD^	0.22 ± 0.02^dE^	0.33 ± 0.1^cdE^
	14	0.59 ± 0.13^fD^	0.69 ± 0.09^fgD^	0.74 ± 0.05^fgD^	1 ± 0.07^eD^
	21	1.32 ± 0.05^iC^	1.71 ± 0.011^ghC^	1.84 ± 0.02^fgC^	2 ± 0.13^efC^
	28	2.28 ± 0.09^jB^	2.65 ± 0.1^hiB^	2.86 ± 0.11^ghB^	3.033 ± 0.41^fgB^
	35	5.9 ± 0.12^iA^	6.02 ± 0.07^hiA^	6.22 ± 0.13^ghA^	6.36 ± 0.12^efA^
Triene conjugates (μmol/g)	0	0.01 ± 0^F^	0.01 ± 0^F^	0.01 ± 0^E^	0.01 ± 0^D^
	7	0.065 ± 0.002^eE^	0.026 ± 0.008^gE^	0.047 ± 0.012^efgE^	0.034 ± 0.001^fgD^
	14	0.17 ± 0.011^ghD^	0.24 ± 0.015^fgD^	0.34 ± 0.023^eD^	0.45 ± 0.017^dC^
	21	0.96 ± 0.03^gC^	1.08 ± 0.008^gC^	1.26 ± 0.017^fC^	1.56 ± 0.033^eB^
	28	2.07 ± 0.04^deB^	2.18 ± 0.1^deB^	2.23 ± 0.036^deB^	2.5 ± 0.023^cdA^
	35	3.51 ± 0.04^gA^	3.75 ± 0.023^efA^	3.86 ± 0.053^efA^	4.26 ± 0.13^dA^
Thiobarbiotic acid (mg MLD/g)	0	0.07 ± 0^E^	0.07 ± 0^D^	0.07 ± 0^E^	0.07 ± 0^E^
	7	0.14 ± 0.03^hE^	0.51 ± 0.1^bcdeD^	0.31 ± 0.032^fgE^	0.4 ± 0.011^defgD^
	14	0.52 ± 0.04^gD^	0.76 ± 0.083^fgD^	0.81 ± 0.02^fD^	1.09 ± 0.081^deD^
	21	1.2 ± 0.09^iC^	1.52 ± 0.04^ghiC^	1.67 ± 0.07f^ghC^	1.79 ± 0.06^fgC^
	28	2.52 ± 0.13^gB^	2.84 ± 0.06^efgB^	2.92 ± 0.19^efgB^	3.01 ± 0.09^efB^
	35	4.36 ± 0.08^hA^	4.64 ± 0.11^ghA^	4.88 ± 0.1f^gA^	5.01 ± 0.1^efA^
Anisidine (Mg/g)	0	0.54 ± 0^D^	0.54 ± 0^E^	0.54 ± 0^D^	0.54 ± 0^E^
	7	0.63 ± 0.1^eD^	0.86 ± 0.083^edE^	0.8 ± 0.03^eD^	0.71 ± 0.1^edE^
	14	1.92 ± 0.16^gC^	2.14 ± 0.13^fgD^	2.17 ± 0.15^fgC^	2.83 ± 0.051^edD^
	21	3.1 ± 0.45^gC^	3.2 ± 0.09^gC^	3.36 ± 0.31^fgC^	4 ± 0.07^degC^
	28	6.02 ± 0.141^fB^	6.37 ± 0.16^eB^	6.43 ± 0.2^deB^	7.03 ± 0.03^cB^
	35	9.13 ± 0.12^hA^	9.63 ± 0.11^hA^	10 ± 0.12^gA^	10.2 ± 0.08^gA^
Totox	0	3.5 ± 0^D^	3.5 ± 0^F^	3.5 ± 0^F^	3.5 ± 0^F^
	7	4.55 ± 0.26^gD^	6.8 ± 0.16^eE^	4.78 ± 0.05^gE^	5.86 ± 0.16^fE^
	14	12.98 ± 0.38^gC^	14 ± 0.34^fgD^	15.49 ± 0.19^fgD^	15.23 ± 0.21^efD^
	21	16.29 ± 0.48^jC^	17.88 ± 0.13^hiC^	18.36 ± 0.52^hC^	19.94 ± 0.35^gC^
	28	24.43 ± 0.22^iB^	26.25 ± 0.3^hgB^	26.63 ± 0.34^gB^	27.75 ± 0.09^fB^
	35	35.79 ± 0.36^hA^	36.64 ± 0.21^hA^	37.96 ± 0.033^gA^	38.3 ± 0.3^fgA^

*Note:* Different lowercase letters indicate a significant difference between rosemary and buckwheat extracts (*p* < 0.05). Different capital letters indicate significant differences between different days (*p* < 0.05).

## 4 Conclusion

The methanolic extracts of buckwheat and rosemary in different concentrations (0 + 800, 200 + 600, 400 + 400, 600 + 200, 800 + 0 ppm) and synthetic antioxidant (50 and 75 mg/kg) were added to soybean oil and were evaluated during storage time using peroxide number, TBA, anisidine number, conjugated DN and TN, and Totox number. The results indicated that both buckwheat and rosemary contain abundant phenolic and flavonoid compounds, with rosemary exhibiting significantly higher levels. In terms of antioxidant activity, rosemary demonstrated greater free‐radical inhibition and iron reduction capacity compared to common buckwheat. Treatments containing 800 ppm rosemary and 800 ppm buckwheat did not show significant differences in all parameters when compared to the synthetic antioxidant at a concentration of 75 ppm and were chosen as sufficient treatments for combining with TBHQ. Treatment of 800 ppm buckwheat + 75 ppm TBHQ demonstrated the most favorable results in terms of oxidation indices. Consequently, it can be concluded that both buckwheat and rosemary can be effectively utilized in soybean oil at a concentration of 800 ppm, either individually or in combination with the synthetic antioxidant TBHQ at concentrations of 50 or 75 ppm. More research on the antioxidant properties of buckwheat and its comparison with other synthetic antioxidants such as BHA, BHT, and PG, as well as the use of other sources of antioxidants such as thyme and comparison with the antioxidant capacity of buckwheat is proposed.

## Author Contributions

Behnam Alizadeh‐Salmani performed the analysis and writing – original draft. Masoumeh Arab and Jalal Sadeghizadeh‐Yazdi had the idea for the article. Mojtaba Yousefi and Sara Jambarsang performed the literature search and data analysis. All authors drafted and/or critically revised the work.

## Conflicts of Interest

The authors declare no conflicts of interest.

## Data Availability

The authors have nothing to report.

## References

[fsn370416-bib-0001] Abada, E. , A. M. Mashlawi , A. A. Gadallah , et al. 2025. “Antimicrobial With Time‐Kill Kinetics, Antioxidant, and Anticancer Properties of *Rosmarinus officinalis* L. Oil Extract Based on Its Bioactive Components.” BioResources 20: 2728–2744.

[fsn370416-bib-0002] Abou Samra, M. , V. S. Chedea , A. Economou , A. Calokerinos , and P. Kefalas . 2011. “Antioxidant/Prooxidant Properties of Model Phenolic Compounds: Part I. Studies on Equimolar Mixtures by Chemiluminescence and Cyclic Voltammetry.” Food Chemistry 125, no. 2: 622–629. 10.1016/j.foodchem.2010.08.076.

[fsn370416-bib-0003] Al‐jaafreh, A. M. 2024. “Evaluation of Antioxidant Activities of Rosemary (*Rosmarinus officinalis* L.) Essential Oil and Different Types of Solvent Extractions.” Biomedical and Pharmacology Journal 17, no. 1: 323–339.

[fsn370416-bib-0004] Amiri, M. , M. Arab , E. K. Sadrabad , N. Mollakhalili‐Meybodi , and H. Fallahzadeh . 2023. “Effect of Gamma Irradiation Treatment on the Antioxidant Activity, Phenolic Compounds and Flavonoid Content of Common Buckwheat.” Radiation Physics and Chemistry 212: 111–127.

[fsn370416-bib-0005] Andrade, J. M. , C. Faustino , C. Garcia , D. Ladeiras , C. P. Reis , and P. Rijo . 2018. “ *Rosmarinus officinalis* L.: An Update Review of Its Phytochemistry and Biological Activity.” Future Science OA 4, no. 4: Fso283. 10.4155/fsoa-2017-0124. 29682318 PMC5905578

[fsn370416-bib-0006] Becerril‐Sánchez, A. L. , B. Quintero‐Salazar , O. Dublán‐García , and H. B. Escalona‐Buendía . 2021. “Phenolic Compounds in Honey and Their Relationship With Antioxidant Activity, Botanical Origin, and Color.” Antioxidants 10, no. 11: 1700. 34829570 10.3390/antiox10111700 PMC8614671

[fsn370416-bib-0007] Benbouriche, A. , H. Haddadi‐Guemghar , M. Bachir‐Bey , et al. 2022. “Improvement of Thermo‐Resistance and Quality of Soybean Oil by Blending With Cold‐Pressed Oils Using Simplex Lattice Mixture Design.” OCL 29: 33.

[fsn370416-bib-0008] Bensid, A. , N. El Abed , A. Houicher , J. M. Regenstein , and F. Özogul . 2022. “Antioxidant and Antimicrobial Preservatives: Properties, Mechanism of Action and Applications in Food–a Review.” Critical Reviews in Food Science and Nutrition 62, no. 11: 2985–3001. 33337242 10.1080/10408398.2020.1862046

[fsn370416-bib-0009] Bhatia, N. K. , V. R. Tomar , Ishika , S. Kishor , and S. Deep . 2022. “Effect of pH and Temperature on Physicochemical Properties, Aggregation Behaviour and Degradation Kinetics of Quercetin and Baicalein in Nearly Aqueous Media.” Journal of Molecular Liquids 366: 120236.

[fsn370416-bib-0010] Bhinder, S. , B. Singh , A. Kaur , et al. 2019. “Effect of Infrared Roasting on Antioxidant Activity, Phenolic Composition and Maillard Reaction Products of Tartary Buckwheat Varieties.” Food Chemistry 285: 240–251. 30797341 10.1016/j.foodchem.2019.01.141

[fsn370416-bib-0011] Bonner, M. Y. , and J. L. Arbiser . 2014. “The Antioxidant Paradox: What Are Antioxidants and How Should They Be Used in a Therapeutic Context for Cancer.” Future Medicinal Chemistry 6, no. 12: 1413–1422. 10.4155/fmc.14.86. 25329197 PMC4412352

[fsn370416-bib-0012] Chammem, N. , S. Saoudi , I. Sifaoui , et al. 2015. “Improvement of Vegetable Oils Quality in Frying Conditions by Adding Rosemary Extract.” Industrial Crops and Products 74: 592–599. 10.1016/j.indcrop.2015.05.054.

[fsn370416-bib-0013] Chen, X. , H. Li , B. Zhang , and Z. Deng . 2022. “The Synergistic and Antagonistic Antioxidant Interactions of Dietary Phytochemical Combinations.” Critical Reviews in Food Science and Nutrition 62, no. 20: 5658–5677. 10.1080/10408398.2021.1888693. 33612011

[fsn370416-bib-0014] Chen, X. , Y. Zhang , Y. Zu , L. Yang , Q. Lu , and W. Wang . 2014. “Antioxidant Effects of Rosemary Extracts on Sunflower Oil Compared With Synthetic Antioxidants.” International Journal of Food Science and Technology 49, no. 2: 385–391.

[fsn370416-bib-0015] Choulitoudi, E. , M. Xristou , D. Tsimogiannis , and V. Oreopoulou . 2021. “The Effect of Temperature on the Phenolic Content and Oxidative Stability of o/w Emulsions Enriched With Natural Extracts From *Satureja thymbra* .” Food Chemistry 349: 129206. 10.1016/j.foodchem.2021.129206. 33578245

[fsn370416-bib-0016] Cordeiro, A. , M. Medeiros , N. Santos , et al. 2013. “Rosemary (*Rosmarinus officinalis* L.) Extract: Thermal Study and Evaluation of the Antioxidant Effect on Vegetable Oils.” Journal of Thermal Analysis and Calorimetry 113: 889–895.

[fsn370416-bib-0017] Di Pietro, M. E. , A. Mannu , and A. Mele . 2020. “NMR Determination of Free Fatty Acids in Vegetable Oils.” Processes 8, no. 4: 410.

[fsn370416-bib-0018] Dias, L. S. , M. E. Menis , and N. Jorge . 2015. “Effect of Rosemary (*Rosmarinus officinalis*) Extracts on the Oxidative Stability and Sensory Acceptability of Soybean Oil.” Journal of the Science of Food and Agriculture 95, no. 10: 2021–2027. 25214375 10.1002/jsfa.6914

[fsn370416-bib-0019] Fernandes, R. P. , M. A. Trindade , F. G. Tonin , et al. 2016. “Evaluation of Antioxidant Capacity of 13 Plant Extracts by Three Different Methods: Cluster Analyses Applied for Selection of the Natural Extracts With Higher Antioxidant Capacity to Replace Synthetic Antioxidant in Lamb Burgers.” Journal of Food Science and Technology 53, no. 1: 451–460. 10.1007/s13197-015-1994-x. 26787964 PMC4711430

[fsn370416-bib-0020] Flores, M. , V. Avendaño , J. Bravo , et al. 2021. “Edible oil parameters during deterioration processes.” International Journal of Food Science 2021, no. 1: 7105170. 34568484 10.1155/2021/7105170 PMC8463213

[fsn370416-bib-0021] Fu, Y. , W. Liu , and O. P. Soladoye . 2021. “Towards Innovative Food Processing of Flavonoid Compounds: Insights Into Stability and Bioactivity.” LWT ‐ Food Science and Technology 150: 111968.

[fsn370416-bib-0022] Geng, L. , K. Liu , and H. Zhang . 2023. “Lipid Oxidation in Foods and Its Implications on Proteins.” Frontiers in Nutrition 10, no. 1: 192–199. 10.3389/fnut.2023.1192199 PMC10307983 37396138

[fsn370416-bib-0023] Gerde, J. , E. Hammond , L. Johnson , C. Su , T. Wanf , and P. White . 2020. “Soybean oil.” In Bailey's Industrial Oil and Fat Products. John Wiley & Sons.

[fsn370416-bib-0024] Gongling, Z. , L. Bing , and G. Yancheng . 2016. “Effect of Rosemary Extract and Tbhq on the Stability of Radish Seed Oil.” Journal of the Chemical Society of Pakistan 38, no. 04: 631–633.

[fsn370416-bib-0025] Gulcin, İ. 2020. “Antioxidants and Antioxidant Methods: An Updated Overview.” Archives of Toxicology 94, no. 3: 651–715. 32180036 10.1007/s00204-020-02689-3

[fsn370416-bib-0026] Gulcin, İ. , and S. H. Alwasel . 2023. “DPPH Radical Scavenging Assay.” Processes 11, no. 8: 2248.

[fsn370416-bib-0027] Guo, Q. , S. Gao , Y. Sun , Y. Gao , X. Wang , and Z. Zhang . 2016. “Antioxidant Efficacy of Rosemary Ethanol Extract in Palm Oil During Frying and Accelerated Storage.” Industrial Crops and Products 94: 82–88. 10.1016/j.indcrop.2016.08.032.

[fsn370416-bib-0028] Hajimehdipoor, H. , R. Shahrestani , and M. Shekarchi . 2014. “Investigating the Synergistic Antioxidant Effects of Some Flavonoid and Phenolic Compounds.” Research Journal of Pharmacognosy 1, no. 3: 35–40.

[fsn370416-bib-0029] Holasova, M. , V. Fiedlerova , H. Smrcinova , M. Orsak , J. Lachman , and S. Vavreinova . 2002. “Buckwheat—The Source of Antioxidant Activity in Functional Foods.” Food Research International 35, no. 2: 207–211. 10.1016/S0963-9969(01)00185-5.

[fsn370416-bib-0030] Hu, W. , G. Y. Sarengaowa , and K. Feng . 2022. “Biosynthesis of Phenolic Compounds and Antioxidant Activity in Fresh‐Cut Fruits and Vegetables.” Frontiers in Microbiology 13: 906069. 35694311 10.3389/fmicb.2022.906069 PMC9176389

[fsn370416-bib-0031] Huyut, Z. , Ş. Beydemir , and İ. Gülçin . 2017. “Antioxidant and Antiradical Properties of Selected Flavonoids and Phenolic Compounds.” Biochemistry Research International 2017, no. 1: 7616791. 29158919 10.1155/2017/7616791 PMC5660747

[fsn370416-bib-0032] Indiarto, R. , and M. A. H. Qonit . 2020. “A Review of Soybean Oil Lipid Oxidation and Its Prevention Techniques.” International Journal of Advanced Science and Technology 29, no. 6: 5030–5037.

[fsn370416-bib-0033] Irakli, M. , A. Skendi , E. Bouloumpasi , S. Christaki , C. G. Biliaderis , and P. Chatzopoulou . 2023. “Sustainable Recovery of Phenolic Compounds From Distilled Rosemary by‐Product Using Green Extraction Methods: Optimization, Comparison, and Antioxidant Activity.” Molecules 28, no. 18: 6669. 10.3390/molecules28186669. 37764444 PMC10537096

[fsn370416-bib-0034] Khoshdouni, F. Z. 2021. “The Effect of Extraction Method (Ultrasonic, Maceration, and Soxhlet) and Solvent Type on the Extraction Rate of Phenolic Compounds and Extraction Efficiency of *Arctium lappa* L. Roots and *Polygonum aviculare* L. Grass.”

[fsn370416-bib-0035] Kreft, I. , M. Germ , A. Golob , B. Vombergar , F. Bonafaccia , and Z. Luthar . 2022. “Impact of Rutin and Other Phenolic Substances on the Digestibility of Buckwheat Grain Metabolites.” International Journal of Molecular Sciences 23, no. 7: 3923. 35409281 10.3390/ijms23073923 PMC8999605

[fsn370416-bib-0036] Kumari Singh, P. , R. Chopra , M. Garg , et al. 2024. “Shelf Life Enhancement of Structured Lipids Rich in Omega‐3 Fatty Acids Using Rosemary Extract: A Sustainable Approach.” ACS Omega 9, no. 29: 31359–31372. 10.1021/acsomega.3c09584. 39072080 PMC11270689

[fsn370416-bib-0037] Lee, J. , C. Boo , S.‐j. Hong , and E.‐C. Shin . 2021. “Chemosensory Device Assisted‐Estimation of the Quality of Edible Oils With Repetitive Frying.” Foods 10, no. 5: 972. 33946677 10.3390/foods10050972 PMC8146517

[fsn370416-bib-0038] Lourenço, S. C. , M. Moldão‐Martins , and V. D. Alves . 2019. “Antioxidants of Natural Plant Origins: From Sources to Food Industry Applications.” Molecules 24, no. 22: 4132. 31731614 10.3390/molecules24224132 PMC6891691

[fsn370416-bib-0039] Mansour, H. M. M. , S. A. El‐Sohaimy , A. M. Zeitoun , and E. M. Abdo . 2022. “Effect of Natural Antioxidants From Fruit Leaves on the Oxidative Stability of Soybean Oil During Accelerated Storage.” Antioxidants 11, no. 9: 1691. 36139765 10.3390/antiox11091691 PMC9495815

[fsn370416-bib-0041] Martin‐Rubio, A. S. , P. Sopelana , and M. D. Guillén . 2020. “Assessment of Soybean Oil Oxidative Stability From Rapid Analysis of Its Minor Component Profile.” Molecules 25, no. 20: 4860. 33096833 10.3390/molecules25204860 PMC7594062

[fsn370416-bib-0040] Martínez, M. L. , M. C. Penci , V. Ixtaina , P. D. Ribotta , and D. Maestri . 2013. “Effect of Natural and Synthetic Antioxidants on the Oxidative Stability of Walnut Oil Under Different Storage Conditions.” LWT‐Food Science and Technology 51, no. 1: 44–50.

[fsn370416-bib-0042] Memon, H. , S. T. Sherazi , S. A. Mahesar , H. Shaikh , and N. Malghani . 2022. “Comparative Study of Quality and Thermo‐Oxidative Stability of Soybean, Palm Olein and Canola Oils With Their Blends.” Authorea Preprints. 10.5650/jos.ess22431 37380483

[fsn370416-bib-0043] Mena, P. , M. Cirlini , M. Tassotti , K. A. Herrlinger , C. Dall'Asta , and D. Del Rio . 2016. “Phytochemical Profiling of Flavonoids, Phenolic Acids, Terpenoids, and Volatile Fraction of a Rosemary (*Rosmarinus officinalis* L.) Extract.” Molecules 21, no. 11: 1576. 10.3390/molecules21111576. 27869784 PMC6273513

[fsn370416-bib-0044] Micić, D. , S. Đurović , P. Riabov , et al. 2021. “Rosemary Essential Oils as a Promising Source of Bioactive Compounds: Chemical Composition, Thermal Properties, Biological Activity, and Gastronomical Perspectives.” Foods 10, no. 11: 2734. 34829014 10.3390/foods10112734 PMC8623706

[fsn370416-bib-0045] Miguel, M. G. 2010. “Antioxidant and Anti‐Inflammatory Activities of Essential Oils: A Short Review.” Molecules 15, no. 12: 9252–9287. 10.3390/molecules15129252. 21160452 PMC6259136

[fsn370416-bib-0046] Moczkowska, M. , S. Karp , O. K. Horbanczuk , M. Hanula , J. Wyrwisz , and M. A. Kurek . 2020. “Effect of Rosemary Extract Addition on Oxidative Stability and Quality of Hemp Seed Oil.” Food and Bioproducts Processing 124: 33–47. 10.1016/j.fbp.2020.08.002.

[fsn370416-bib-0047] Mollica, F. , M. Lucarini , C. Passerini , et al. 2020. “Effect of Antioxidants on High‐Temperature Stability of Renewable Bio‐Oils Revealed by an Innovative Method for the Determination of Kinetic Parameters of Oxidative Reactions.” Antioxidants 9, no. 5: 399. 32397271 10.3390/antiox9050399 PMC7278824

[fsn370416-bib-0048] Naksuriya, O. , and S. Okonogi . 2015. “Comparison and Combination Effects on Antioxidant Power of Curcumin With Gallic Acid, Ascorbic Acid, and Xanthone.” Drug Discoveries and Therapeutics 9, no. 2: 136–141. 10.5582/ddt.2015.01013. 25994066

[fsn370416-bib-0049] Neunert, G. , P. Górnaś , K. Dwiecki , A. Siger , and K. Polewski . 2015. “Synergistic and Antagonistic Effects Between Alpha‐Tocopherol and Phenolic Acids in Liposome System: Spectroscopic Study.” European Food Research and Technology 241: 749–757.

[fsn370416-bib-0050] Nieto, G. , G. Ros , and J. Castillo . 2018. “Antioxidant and Antimicrobial Properties of Rosemary ( *Rosmarinus officinalis* , L.): A Review.” Medicines (Basel) 5, no. 3: 98. 10.3390/medicines5030098. 30181448 PMC6165352

[fsn370416-bib-0051] Nitbani, F. O. , P. J. P. Tjitda , B. A. Nurohmah , and H. E. Wogo . 2020. “Preparation of Fatty Acid and Monoglyceride From Vegetable Oil.” Journal of Oleo Science 69, no. 4: 277–295. 32249258 10.5650/jos.ess19168

[fsn370416-bib-0053] Olszowy‐Tomczyk, M. 2020. “Synergistic, Antagonistic and Additive Antioxidant Effects in the Binary Mixtures.” Phytochemistry Reviews 19, no. 1: 63–103. 10.1007/s11101-019-09658-4.

[fsn370416-bib-0052] Olszowy, M. , A. L. Dawidowicz , and M. Jóźwik‐Dolęba . 2019. “Are Mutual Interactions Between Antioxidants the Only Factors Responsible for Antagonistic Antioxidant Effect of Their Mixtures? Additive and Antagonistic Antioxidant Effects in Mixtures of Gallic, Ferulic and Caffeic Acids.” European Food Research and Technology 245: 1473–1485.

[fsn370416-bib-0054] Padehban, L. , S. Ansari , and R. Koshani . 2018. “Effect of Packaging Method, Temperature and Storage Period on Physicochemical and Sensory Properties of Wild Almond Kernel.” Journal of Food Science and Technology 55, no. 9: 3408–3416. 10.1007/s13197-018-3239-2. 30150799 PMC6098806

[fsn370416-bib-0055] Palma, A. , M. Ruiz Montoya , M. J. Díaz , J. F. Arteaga , R. Estévez Brito , and J. M. Rodríguez Mellado . 2017. “Evaluation of Synergistic and Antagonistic Effects Between Some Selected Antioxidants by Means of an Electrochemical Technique.” International Journal of Food Science and Technology 52: 1639–1644.

[fsn370416-bib-0056] Parcheta, M. , R. Świsłocka , S. Orzechowska , M. Akimowicz , R. Choińska , and W. Lewandowski . 2021. “Recent Developments in Effective Antioxidants: The Structure and Antioxidant Properties.” Materials 14, no. 8: 1984. 33921014 10.3390/ma14081984 PMC8071393

[fsn370416-bib-0057] Peyrat‐Maillard, M. N. , M. E. Cuvelier , and C. Berset . 2003. “Antioxidant Activity of Phenolic Compounds in 2,2′‐Azobis (2‐Amidinopropane) Dihydrochloride (AAPH)‐Induced Oxidation: Synergistic and Antagonistic Effects.” Journal of the American Oil Chemists' Society 80, no. 10: 1007–1012. 10.1007/s11746-003-0812-z.

[fsn370416-bib-0058] Poljsak, B. , V. Kovač , and I. Milisav . 2021. “Antioxidants, Food Processing and Health.” Antioxidants 10, no. 3: 433. 33799844 10.3390/antiox10030433 PMC8001021

[fsn370416-bib-0059] Ravi Kiran, C. , I. Sasidharan , D. R. Soban Kumar , and A. Sundaresan . 2015. “Influence of Natural and Synthetic Antioxidants on the Degradation of Soybean Oil at Frying Temperature.” Journal of Food Science and Technology 52, no. 8: 5370–5375. 10.1007/s13197-015-1774-7. 26243968 PMC4519514

[fsn370416-bib-0060] Rocío Teruel, M. , M. D. Garrido , M. C. Espinosa , and M. B. Linares . 2015. “Effect of Different Format‐Solvent Rosemary Extracts (*Rosmarinus officinalis*) on Frozen Chicken Nuggets Quality.” Food Chemistry 172: 40–46. 10.1016/j.foodchem.2014.09.018. 25442521

[fsn370416-bib-0061] Rojas‐Ocampo, E. , L. Torrejón‐Valqui , L. D. Muñóz‐Astecker , M. Medina‐Mendoza , D. Mori‐Mestanza , and E. M. Castro‐Alayo . 2021. “Antioxidant Capacity, Total Phenolic Content and Phenolic Compounds of Pulp and Bagasse of Four Peruvian Berries.” Heliyon 7, no. 8: e07787. 34430752 10.1016/j.heliyon.2021.e07787 PMC8367789

[fsn370416-bib-0062] Roshni, A. 2019. “Comparison of Chemical Characterises of Crude and Refined Edible Vegetable Oils.” Paper presented at the AIP Conference Proceedings.

[fsn370416-bib-0063] Sadowska‐Bartosz, I. , and G. Bartosz . 2022. “Evaluation of the Antioxidant Capacity of Food Products: Methods, Applications and Limitations.” Processes 10, no. 10: 2031.

[fsn370416-bib-0064] Sahunie, A. 2024. “Effect of *Rosmarinus officinalis* and *Origanum majorana* Extracts on Stability of Sunflower Oil During Storage and Repeated Heating.” Oil Crop Science 9, no. 1: 29–37. 10.1016/j.ocsci.2023.12.006.

[fsn370416-bib-0065] Salehi, A. , S. Fallah , H.‐P. Kaul , and K. Zitterl‐Eglseer . 2018. “Antioxidant Capacity and Polyphenols in Buckwheat Seeds From Fenugreek/Buckwheat Intercrops as Influenced by Fertilization.” Journal of Cereal Science 84: 142–150.

[fsn370416-bib-0066] Samotyja, U. , and M. Małecka . 2010. “Antioxidant Activity of Blackcurrant Seeds Extract and Rosemary Extracts in Soybean Oil.” European Journal of Lipid Science and Technology 112, no. 12: 1331–1336. 10.1002/ejlt.201000042.

[fsn370416-bib-0067] Sethi, S. , A. Joshi , B. Arora , A. Bhowmik , R. Sharma , and P. Kumar . 2020. “Significance of FRAP, DPPH, and CUPRAC Assays for Antioxidant Activity Determination in Apple Fruit Extracts.” European Food Research and Technology 246: 591–598.

[fsn370416-bib-0068] Shahidi, F. , and A. Hossain . 2022. “Role of Lipids in Food Flavor Generation.” Molecules 27, no. 15: 5014. 35956962 10.3390/molecules27155014 PMC9370143

[fsn370416-bib-0069] Sharma, Y. , R. Velamuri , J. Fagan , and J. Schaefer . 2020. “Full‐Spectrum Analysis of Bioactive Compounds in Rosemary ( *Rosmarinus officinalis* L.) as Influenced by Different Extraction Methods.” Molecules 25, no. 20: 4599. 10.3390/molecules25204599. 33050282 PMC7587196

[fsn370416-bib-0070] Shen, N. , T. Wang , Q. Gan , S. Liu , L. Wang , and B. Jin . 2022. “Plant Flavonoids: Classification, Distribution, Biosynthesis, and Antioxidant Activity.” Food Chemistry 383: 132–531. 10.1016/j.foodchem.2022.132531 35413752

[fsn370416-bib-0071] Shi, L. , W. Zhao , Z. Yang , V. Subbiah , and H. A. R. Suleria . 2022. “Extraction and Characterization of Phenolic Compounds and Their Potential Antioxidant Activities.” Environmental Science and Pollution Research 29, no. 54: 81112–81129. 36201076 10.1007/s11356-022-23337-6 PMC9606084

[fsn370416-bib-0072] Sik, B. , E. Hanczné Lakatos , V. Kapcsándi , R. Székelyhidi , and Z. Ajtony . 2021. “Investigation of the Long‐Term Stability of Various Tinctures Belonging to the Lamiaceae Family by HPLC and Spectrophotometry Method.” Chemical Papers 75, no. 11: 5781–5791. 10.1007/s11696-021-01755-z.

[fsn370416-bib-0073] Singh, B. , S. Oberoi , and A. Kaur . 2024. “Phenolic Composition, Antioxidant Activity and Health Benefits of Tartary (*Fagopyrum tataricum* Gaerth) and Common (*F. esculentum* Moench) Buckwheat Grains: A Review.” Food Chemistry Advances 100: 820.

[fsn370416-bib-0074] Skroza, D. , V. Šimat , L. Vrdoljak , et al. 2022. “Investigation of Antioxidant Synergisms and Antagonisms Among Phenolic Acids in the Model Matrices Using FRAP and ORAC Methods.” Antioxidants 11, no. 9: 1784. 10.3390/antiox11091784. 36139858 PMC9495677

[fsn370416-bib-0075] Srivastava, Y. , and A. D. Semwal . 2015. “A Study on Monitoring of Frying Performance and Oxidative Stability of Virgin Coconut Oil (VCO) During Continuous/Prolonged Deep Fat Frying Process Using Chemical and FTIR Spectroscopy.” Journal of Food Science and Technology 52, no. 2: 984–991. 10.1007/s13197-013-1078-8. 25694709 PMC4325013

[fsn370416-bib-0076] Subroto, E. , A. D. Pangawikan , V. P. Yarlina , and N. F. Isnaeni . 2020. “Characteristics, Purification, and the Recent Applications of Soybean Oil in Fat‐Based Food Products: A Review.” International Journal 8, no. 7: 3003–3011.

[fsn370416-bib-0077] Sun, T. , and C.‐T. Ho . 2005. “Antioxidant Activities of Buckwheat Extracts.” Food Chemistry 90, no. 4: 743–749. 10.1016/j.foodchem.2004.04.035.

[fsn370416-bib-0078] Tan, C. H. , C. J. Lee , S. N. Tan , D. T. S. Poon , C. Y. E. Chong , and L. P. Pui . 2021. “Red Palm Oil: A Review on Processing, Health Benefits and Its Application in Food.” Journal of Oleo Science 70, no. 9: 1201–1210. 34373407 10.5650/jos.ess21108

[fsn370416-bib-0079] Tavadyan, L. A. , and S. H. Minasyan . 2019. “Synergistic and Antagonistic Co‐Antioxidant Effects of Flavonoids With Trolox or Ascorbic Acid in a Binary Mixture.” Journal of Chemical Sciences 131: 40.

[fsn370416-bib-0080] Tohma, S. , and S. Turan . 2015. “Rosemary Plant (*Rosmarinus officinalis* L.), Solvent Extract and Essential Oil Can Be Used to Extend the Usage Life of Hazelnut Oil During Deep Frying.” European Journal of Lipid Science and Technology 117, no. 12: 1978–1990. 10.1002/ejlt.201400382.

[fsn370416-bib-0081] Topal, M. , and İ. Gulcin . 2022. “Evaluation of the In Vitro Antioxidant, Antidiabetic and Anticholinergic Properties of Rosmarinic Acid From Rosemary (*Rosmarinus officinalis* L.).” Biocatalysis and Agricultural Biotechnology 43: 102–417.

[fsn370416-bib-0082] Vicol, C. , A. Sârghi , A. Fifere , and G. Duca . 2024. “Antioxidant Co‐Actions of Ascorbic and Dihydroxyfumaric Acids Investigated by EPR Spectroscopy.” Chemistry Journal of Moldova 19: 29–36.

[fsn370416-bib-0083] Wang, W. , C. Sun , L. Mao , et al. 2016. “The Biological Activities, Chemical Stability, Metabolism and Delivery Systems of Quercetin: A Review.” Trends in Food Science and Technology 56: 21–38. 10.1016/j.tifs.2016.07.004. PMC7127391 32288229

[fsn370416-bib-0084] Wann, A. I. , B. C. Percival , K. Woodason , M. Gibson , S. Vincent , and M. Grootveld . 2021. “Comparative 1H NMR‐Based Chemometric Evaluations of the Time‐Dependent Generation of Aldehydic Lipid Oxidation Products in Culinary Oils Exposed to Laboratory‐Simulated Shallow Frying Episodes: Differential Patterns Observed for Omega‐3 Fatty Acid‐Containing Soybean Oils.” Food 10, no. 10: 2481. 10.3390/foods10102481 PMC8535530 34681530

[fsn370416-bib-0085] Wigati, D. , L. Setyaningrum , and D. K. Pratoko . 2023. “The Effect of Extraction Methods on the Total Phenols and Total Flavonoids Content of Jackfruit (*Artocarpus heterophyllus* Lamk) Peels Extract.” EKSAKTA: Berkala Ilmiah Bidang MIPA 24, no. 01: 30–39.

[fsn370416-bib-0086] Wojeicchowski, J. P. , A. M. Ferreira , D. O. Abranches , M. R. Mafra , and J. A. Coutinho . 2020. “Using COSMO‐RS in the Design of Deep Eutectic Solvents for the Extraction of Antioxidants From Rosemary.” ACS Sustainable Chemistry and Engineering 8, no. 32: 12132–12141.

[fsn370416-bib-0087] Yasin, F. M. , Z. Zam Zam , and K. A. Rakhman . 2022. “Analysis of Antioxidant Content of Anthocyanin in the Lobi‐Lobi Fruit (*Flacourtian inermis*) and Jamblang Fruit (*Syzygium cumini* L Skeel) Using the Dpph Method With Spectrophotometry.” Jurnal Biosains Pascasarjana 24: 8–14.

[fsn370416-bib-0088] Zahran, H. , and Z. Najafi . 2020. “Enhanced Stability of Refined Soybean Oil Enriched With Phenolic Compounds of Olive Leaves.” Egyptian Journal of Chemistry 63, no. 1: 215–224.

[fsn370416-bib-0089] Zeb, A. 2020. “Concept, Mechanism, and Applications of Phenolic Antioxidants in Foods.” Journal of Food Biochemistry 44, no. 9: e13394. 32691460 10.1111/jfbc.13394

[fsn370416-bib-0090] Zhang, D. , X. Li , Z. Zhang , et al. 2022. “Influence of Roasting on the Physicochemical Properties, Chemical Composition and Antioxidant Activities of Peanut Oil.” LWT ‐ Food Science and Technology 154: 112–613.

[fsn370416-bib-0091] Zhang, Y. , J. P. Smuts , E. Dodbiba , R. Rangarajan , J. C. Lang , and D. W. Armstrong . 2012. “Degradation Study of Carnosic Acid, Carnosol, Rosmarinic Acid, and Rosemary Extract ( *Rosmarinus officinalis* L.) Assessed Using HPLC.” Journal of Agricultural and Food Chemistry 60, no. 36: 9305–9314. 10.1021/jf302179c. 22881034

[fsn370416-bib-0092] Zhang, Y. , M. Wang , X. Zhang , et al. 2023. “Mechanism, Indexes, Methods, Challenges, and Perspectives of Edible Oil Oxidation Analysis.” Critical Reviews in Food Science and Nutrition 63, no. 21: 4901–4915. 34845958 10.1080/10408398.2021.2009437

[fsn370416-bib-0093] Zhao, Q. , Y. Xu , and Y. Liu . 2022. “Soybean Oil Bodies: A Review on Composition, Properties, Food Applications, and Future Research Aspects.” Food Hydrocolloids 124: 107–296.

[fsn370416-bib-0094] Zhong, L. , Y. Lin , C. Wang , et al. 2022. “Chemical Profile, Antimicrobial and Antioxidant Activity Assessment of the Crude Extract and Its Main Flavonoids From Tartary Buckwheat Sprouts.” Molecules 27, no. 2: 374. 35056695 10.3390/molecules27020374 PMC8779668

[fsn370416-bib-0095] Zhu, F. 2021. “Buckwheat Proteins and Peptides: Biological Functions and Food Applications.” Trends in Food Science and Technology 110: 155–167.

[fsn370416-bib-0096] Zubairee, K. , H. Yalcin , and T. Dursun Capar . 2025. “Sunflower Oil‐Soybean Wax Oleogel: An Oxidation Stable Alternative to Traditional Frying Methods for Doughnut.” Journal of the American Oil Chemists' Society 102, no. 2: 239–250.

